# Prevention of cardiovascular diseases

**DOI:** 10.1186/s12916-015-0507-0

**Published:** 2015-10-12

**Authors:** F. D. Richard Hobbs

**Affiliations:** Nuffield Department of Primary Care Health Sciences, University of Oxford, New Radcliffe House, Radcliffe Observatory Quarter, Walton Street, Oxford, OX2 6GG UK; Harris Manchester College, University of Oxford, Mansfield Road, Oxford, OX1 3TD UK

**Keywords:** Cardiovascular disease, Prevention, Risk factors

## Abstract

Cardiovascular disease (CVD) is the most important cause of premature death and disability globally. Much is known of the main aetiological risk factors, including elevated blood pressure, dyslipidaemia and smoking, with a raft of additional risks of increasing prevalence, such as obesity and diabetes. Furthermore, some of the most secure evidence-based management strategies in healthcare relate to interventions that modify risk. Yet major gaps remain in the implementation of such evidence, summarized in international guideline recommendations. Some of this gap relates to knowledge deficits amongst clinicians, but also to continued uncertainties over interpretation of the evidence base and areas where data are less available. This article collection in *BMC Medicine* seeks to offer reflections in each of these areas of uncertainty, spanning issues of better diagnosis, areas of controversy and glimpses of potentially potent future interventions in the prevention of CVD.

## Editorial

Cardiovascular disease (CVD) remains the leading cause of global morbidity and mortality [1]. The risk factors of abnormal lipids, smoking, hypertension, diabetes, abdominal obesity, poor diet and irregular physical exercise account for more than 90 % of the CVD risk in epidemiological studies [2].

The commonest risk factor is hypertension, with a global prevalence estimated at 26.4 % (972 million adults) in 2000 and a predicted rise of 60 % to a total of 1.56 billion adults (29.2 %) by 2025 [3]. A major predictor for coronary heart disease (CHD) and stroke [4, 5], international guidelines highlight the management of hypertension [6, 7] based on huge clinical outcome trial datasets [8], which show that a net blood pressure (BP) reduction of 10–12 mmHg systolic BP and 5–6 mmHg diastolic BP reduces stroke incidence by 38 % and CHD by 16 % [9]. In absolute terms, treating 1000 patients in four 5-year CVD risk groups observed in the placebo arms of trials (5-year risks of <11 %, 11–15 %, 15–21 % and >21 %) with BP-lowering treatment for 5 years would prevent 14 (95 % CI: 8–21), 20 (95 % CI: 8–31), 24 (95 % CI: 8–40) and 38 (95 % CI: 16–61) cardiovascular events, respectively (*P* = 0.04 for trend) [9].

Interventions that lower low-density lipoprotein cholesterol (LDL-C) concentrations are also proven to significantly reduce the incidence of CHD and other major vascular events in a wide range of individuals. A meta-analysis of 14 statin trials showed that for every 40 mg/dL (1 mmol/L) decrease in LDL-C, it led to a 21 % decrease in CHD risk after 1 year of treatment [10]. These data were incorporated into clinical guidance, such as the American College of Cardiology/American Heart Association (ACC/AHA) [11] and National Cholesterol Education Program (NCEP) Adult Treatment Panel (ATP) III guidelines [12] in the US; the Joint Task Force of the European Society of Cardiology and Other Societies on Cardiovascular Disease Prevention in Clinical Practice guidelines in Europe [13]; and the National Institute for Health and Care Excellence (NICE) in the UK, which all recognise the importance of dyslipidaemia, as well as hypertension and smoking, as the main risk factors for CVD. They also provide practical tools (Framingham, Systematic Coronary Risk Evaluation (SCORE) and QRISK 10-year CVD risk algorithms, respectively) to assist short-term risk estimation in individuals without prior CVD, although there remain many barriers to guideline implementation in routine clinical practice [14].

However, despite this huge evidence base on the aetiology of CVDs and their treatment options, many questions still remain unanswered. Some of these are considered in this special article collection in *BMC Medicine*, including critical reviews on diagnosing hypertension [15], the potential of PCSK9 antibodies [16], an entirely new class of LDL-C modifiers developed from basic concept to phase III trials in less than a decade, and the evidence for smoking reduction interventions [17].

In the near future, there will also be an upcoming forum debate on the relative impact of statins on vascular disease — over 20 years after their introduction and now one of the most prescribed drugs in the world, there remains much debate on these agents. The article collection will also present the updated guidance on stroke prevention in atrial fibrillation (SPAF) which, alongside detection and management of hypertension, is the most important strategy to prevent stroke. Atrial fibrillation (AF) is the commonest cardiac arrhythmia, with about 1–2 % of the general population estimated to be affected [18]. It is a particularly common disorder in older people, with over 5 % over the age of 65 years suffering from AF and around 10 % of people over the age of 75 years [19, 20], with the prevalence predicted to rise [21, 22]. Patients with AF are at an almost five-fold higher risk of stroke compared to age-matched individuals with normal sinus rhythm [23], as well as at a twice as high risk of all-cause mortality and heart failure. About 20 % of all ischaemic strokes are attributable to embolism as a result of AF [24]. Not only do patients with AF have more strokes, they also develop more recurrent strokes, more severe strokes, regardless of age [25], and are more likely to be left with long-term disability and require long-term care [26]. It is a very important topic for patients and for healthcare system payers.

Accompanied by peer-reviewed research papers [27–30], this article collection, *Prevention of cardiovascular diseases*, should be of interest to all *BMC Medicine* readers.

## Abbreviations

ACC: American College of Cardiology
AF: Atrial fibrillation
AHA: American Heart Association
ATP: Adult Treatment Panel
BP: Blood pressure
CHD: Coronary heart disease
CVD: Cardiovascular disease
LDL-C: Low-density lipoprotein cholesterol
NCEP: National Cholesterol Education Program
NICE: National Institute for Health and Care Excellence
SCORE: Systematic Coronary Risk Evaluation
SPAF: Stroke prevention in atrial fibrillation
